# Supplementary material for: Culture is critical in driving orangutan diet development past individual potentials
Source: Nat Hum Behav. 2025 Nov 24;10(2):243–54. doi: 10.1038/s41562-025-02350-y (PMC12932104; doi:10.1038/s41562-025-02350-y)
Supplement: Supplementary file 1 — Supplementary information, including all supplementary text and Supplementary Tables 1–5. [file 41562_2025_2350_MOESM1_ESM.pdf]

nature human behaviour

Supplementary information https://doi.org/10.1038/s41562-025-02350-y

Culture is critical in driving orangutan diet development past individual potentials

In the format provided by the authors and unedited

Table of Contents: Supplementary Information

Section 1: ABM Model Design **Page 2**

1.1 Estimates from wild data: implicit assumptions Page 2

1.2 ABM design choices: implicit assumptions Page 6

Section 2: Statistical analyses **Page 8**

2.1 Estimating parameters using data from wild orangutans at Suaq Page 8

2.2 ABM Output Page 10

Section 3: Supplementary Tables **Page 12**

Table S1. ABM coefficients for the probability of simulated immatures entering each social state when in a given feeding patch, and their subsequent probability of exploring, at different ages. Page 12

Table S2. Summaries of models characterizing the foraging behaviors of adult orangutans at Suaq, including mothers. Page 13

Table S3. Summaries of models used to estimate the probability of immatures being in different social states across development. Page 14

Table S4. Summaries of models used to characterize the relationships between immatures' ages, social states, and their resultant effects on the probability of exploration. Page 15

Table S5. Summaries of models used to characterize the outputs of the ABM. Page 16

Section 4: Supplementary references **Page 17**

Section 1: ABM Model Design

We designed our agent-based model to closely reflect the day-to-day foraging behaviors of wild orangutans. Like all models, our model is a simplification of real-world processes[1,2]. However, our longitudinal observations of orangutan foraging behaviors (including across development) provide a means to design a biologically-informed ABM, in a suitably constrained strategy space, to explore questions surrounding the effects of social learning on diet-repertoire development. As such, our ABM was entirely calibrated using wild data. Despite calibration with longitudinal data, some of our estimates inevitably contained implicit, unavoidable assumptions (see below). Ultimately, for models to be useful tools in characterizing real-world phenomena, assumptions and careful simplifications are always required[1,2]. We demonstrate that whilst our model is a simplification of reality, it captures the outcomes of real-world processes with high accuracy, as identified through validating its outputs using real-world data[1,2]. However, to enshrine principles of open science and transparent reporting, we herein provide further information about the assumptions we make when:

[1] estimating coefficients for our ABM using data collected from the wild,

[2] designing the algorithmic processes of our ABM,

as well as any limitations imposed by these decisions. As mentioned in the Discussion section of our manuscript, many design features of our ABM likely lead to conservative estimates for the relative importance of different social learning mechanisms for diet development. We explain the rationale behind several of these design features, as well as their implicit assumptions and possible limitations within the Methods, Results, and Discussion of our main manuscript. The additional information provided here will not repeat discussions of these points in the main manuscript. Instead, we discuss the nature and possible impact of several additional assumptions and limitations on the conclusions that can be drawn from our ABM. However, in all instances, we believe that it is highly unlikely that the main claims of our manuscript would be affected.

1.1 Estimates from wild data: implicit assumptions

We assume all adult orangutans are aware of all available foods at Suaq

We estimated the total number of available foods at Suaq by counting the number of different foods orangutans ate across all follows ($N_{Follows}$ = 1620; N_{Adults} = 95; N_{Scans} = 402,082). This measure is, more accurately, the number of items in the environment that have been collectively identified as 'edible' by all sampled orangutans. It is likely that orangutans at Suaq have found most of their available foods, given that the Suaq orangutans have likely lived in this ecology for many generations. However, it is possible that a small number of undiscovered foods could exist at Suaq, which could still be discovered during general exploration of immatures and adults.

If wild orangutans identified new foods that the entire population was thus far naïve to, this could theoretically enable individuals to expand their diet repertoires asocially by a small amount. However, within the ABM, we do not include the possibility that orangutans will be able to identify foods that are not eaten by any adult. In reality, consuming completely new items in the environment is risky, given that many species at Suaq (and at other orangutan populations) are toxic. The threat of consuming toxic foods is something which is frequently discussed in relation to reintroduction of captive-born or rehabilitated of primates back into the

wild, thus is a non-negligible threat[3–5]. Secondly, it is not possible to estimate the number of edible, yet unknown foods available to wild orangutans, without being able to confirm whether orangutans can indeed consume each candidate food (which can only be confirmed through observation of consumption). Indeed, identifying available, yet unconsumed foods may be possible by identifying foods that are consumed in other orangutan populations, but not at Suaq, despite being present both study areas. However, for every item that is present at Suaq that is consumed at another orangutan population, it is also consumed by individuals at Suaq. This suggests that the Suaq orangutans have a comprehensive knowledge of available food items.

For these reasons, we restrict our ABM to only consider how social learning influences the rate at which orangutans learn how to eat food items that have been confirmed as edible from wild data, and assume that there are no additional, novel foods in the environment that could be included in diet repertoires through independent exploration.

We do not account for short-term temporal variation in the availability of foods

We estimated the probability of encountering each food item in a given feeding patch by estimating a probability of encountering different foods across each day at Suaq. This distribution was estimated by dividing the frequency that each food item occurred across all follows by the sum total of frequencies across all food items (see Methods). This distribution does not account for temporal heterogeneity in the availability of different food items over development. In reality, foods are often available at specific locations during limited periods of time, such as when trees are fruiting. This may lead to high variance in the intervals between successive visits to feeding patches containing the same food, which is not captured within our ABM. Our ABM therefore assumes that immatures can remember previous explorations, regardless of the time interval which has passed between explorations (see section *Simulated immatures have perfect memory*).

Whilst our model may not accurately capture the heterogeneity of available foods, over long time periods (i.e. several years) the encounter rate for each food item averages out to encounter rates observed in the wild (see associated code for verification of this outcome). Therefore, our estimates of relative food availability still accurately approximate opportunities for simulated immatures to learn about different foods across extended timescales. Future research should examine how this temporal heterogeneity influences immature feeding behaviors after the onset of independence, particularly in instances where independent immatures have smaller diet repertoires. This will provide further insight into how diet repertoire composition and food availability combine to influence the fitness of immatures following independence.

We assume any individual associated with an immature is knowledgeable about available food items

When estimating whether an individual is in close proximity to another conspecific, or is peering at a conspecific's behavior, we include any and all associated individuals in this category. Whilst dependent immatures consistently range with their mothers, other individuals may associate with mother-offspring dyads for short intervals. Additionally, following the onset of independence, immatures can associate with unrelated individuals for short time periods. Particularly towards the end of dependency, immatures exhibit detectable interest in unrelated individuals when in association[6], and therefore it is possible that these individuals can also be the target of learning. Moreover, whilst diets of orangutans at Suaq do vary, their compositions overlap heavily, meaning that most individuals are knowledgeable about available foods, and thus likely offer useful models for immatures to learn from[6,7]. Therefore, when estimating the probability of being in association with a conspecific (including close and distance association) and the probability of peering at conspecifics, we included data on association and peering between immatures any other orangutans, whose interactions with immatures can likely also facilitate knowledge acquisition.

We do not account for wild immatures' existing knowledge when estimating the likelihood that they will peer at or explore encountered food items

We estimate the probability that wild immatures peer at food items, and explore food items (including when not peering) across development (see Methods). We estimated these probabilities using long-term cross-sectional data, and used variation in the ages of wild orangutans to estimate how exploration and peering probabilities are influenced by development (whilst controlling for repeated measures of the same individuals over time).

Because each orangutan at Suaq is not followed every day, it is not possible to determine precisely which food items are known to wild immatures at any given time (unless there is obvious evidence, e.g. feeding on foods without exploration suggests that these foods are known to an individual). Therefore, it was not always possible for us to control for how immatures' existing knowledge may influence the likelihood that they peered at, or explored, encountered food items. In theory, peering and exploration may be less likely to occur if an individual is already aware of a specific food item, and how to process and consume it. Reciprocally, immatures may have higher exploration and peering rates at each age when encountering food items which they are naïve to.

This limitation may influence the results of our model, as we may underestimate the probability that simulated immatures peer at or explore unknown food items. However, we are limited in the extent to which we can adjust our estimations based on these facts. Instead, we used the only data available to us: the age-dependent average peering and exploration rates performed by wild immatures across contexts.

Previous research of orangutan behavioral development reveals that exploration and peering rates follow predictable trajectories across different environments, though certain environmental factors can exacerbate the rates at which both behaviors are performed[8–11]. These developmental trajectories are similar to what we estimated using data from Suaq: exploration & peering rates are higher earlier in life, suggesting that immatures experience a higher drive to perform these behaviors earlier during development. Whilst we cannot account for the effect of existing knowledge on exploration and peering rates, the compatibility of our results and the existing literature suggests that, by leveraging average age-dependent peering and exploration rates, we capture the key dynamics of exploration and peering rates during

development. Further research is needed to understand how existing knowledge influences the rate of exploration and peering in wild immature individuals.

We could not control for the effects of cognitive or physical development

Through peering and exploration, wild immatures may learn about the availability of food items, and perhaps even information about how to perform necessary food processing behaviors. However, following instances of information acquisition through peering, or information generation through exploration, immatures may be prevented from successfully performing feeding behaviors, as further cognitive or physical development is still required[12]. This developmental lag would mean that, when encountering certain foods, immatures may continue to explore these foods, and peer at other individuals when processing the same food, in an attempt to perform the relevant food processing behavior, but without actually learning any new information about food items and their associated processing. The successful processing of these food items may then occur later in time, once the necessary cognitive or physical development has taken place. These developmental constraints likely have more profound implications for foods that involve more complex processing behaviors, or require immatures to meet a threshold of strength or dexterity.

These developmental delays may cause us to overestimate the number of explorations immatures require to learn about how to process food items. In tandem, this may therefore lead to an overestimation of the importance of social learning as a means to upregulate exploration and ultimately learning. We could not explicitly estimate whether physical or cognitive development modulates diet-repertoire learning in wild orangutans from our observational data. However, given that the majority of orangutan diets at Suaq are made of relatively simple foraging behaviors, which can likely be performed with simple forms of cognition, strength and dexterity, it is unlikely that the majority of the diet is impeded by developmental constraints (% of diet that requires 2 or fewer processing steps = 79.8%; diet composition for each complexity category: 0 = 42.4%, 1 = 23.7%, 2 = 13.7%; >2 = 20.2% see Methods for further information about food complexity categories). Thus, for wild orangutans at Suaq, the effects of physical and cognitive development on broad-scale diet development may be minimal; however, the effects of developmental delays on diet learning should be investigated further, where possible.

***The number of required explorations for more complex foods (complexity level 3 and above) are estimated via extrapolation*.**

We estimated the number of times wild immatures explore foods of varying complexity prior to adding them to their diets (see Methods section 'Number of explorations needed to learn'). For more complex foods (level 3-5) we extrapolated the number of required explorations using a linear trend estimated for simpler foods (complexity levels 0-2). This extrapolation was necessary as more complex foods are rarely encountered, thus the number of times wild individuals encounter them prior to learning is challenging to estimate from long-term cross-sectional follow data.

Given that complex foods are more challenging to learn (as more information must be acquired during exploration), and rarely encountered (thus, information may be forgotten between explorations), it may be that these foods require many more explorations to learn than simpler foods that are encountered more regularly. As such, the linear extrapolation we used to infer the number of explorations is potentially a conservative estimate of the number required to learn complex foods.

In such a case, our ABM is likely again conservative in its conclusions for the importance of social learning for diet development. If more complex foods require a greater

number of explorations to be added to diet repertoires, the importance of social learning for upregulating exploration may be even more pronounced for wild individuals. This includes the need to capitalize on limited opportunities for information acquisition about complex resources. Indeed, studies of wild orangutan peering find that immatures peer more often and complex and rarely encountered foods, suggesting that this is indeed the case[8]. We therefore believe that this extrapolation does not influence the conclusions of our study, and leaves room for further examination of the importance of social learning for acquiring complex food processing skills.

We set the exploration rate when simulated immatures are 'alone' to be equal to when they are in 'distant association'.

As immatures are never alone during the dependency period, we could not directly estimate exploration rates when wild dependent immatures were not associated with other individuals. We therefore set their exploration rates when alone to be the same as when in 'distant association' at each age (between 10-50 m away from every other conspecific). Previous research on wild orangutans of older age classes (i.e. adults) has revealed that being in association leads to slightly higher exploration rates than when alone[13]. Thus, our model may somewhat overestimate the likelihood of exploration when immatures are alone. Ultimately, this would make our model's baseline condition (*Exposure Only*) a conservative estimate of diet development without peering and enhancement, as lower exploration rates when alone – as well as the reported exploration rates in distant association – would lead to slower diet development, and would further emphasize the need for social learning to accelerate diet development to meet developmental milestones.

1.2 ABM design choices: implicit assumptions

Simulated immatures do not starve to death

Our model quantified how different forms of social learning influence long-term diet-repertoire development. We did not, however, look at the resultant effects of diet-repertoire development on immature survivability during the dependency and independent phase.

Estimating whether slower diet-repertoire development would reduce calorie intake, and consequently immature survivability, is challenging. This is particularly difficult during the dependency phase, when dependent immatures are energetically supplemented by their mothers through nursing, including up until ages that are close to the onset of independence. Following independence, the effects of reduced diet size on survivability could be evaluated; however, this would require more detailed information surrounding the availability of foods over time, the rates at which foods can be processed and consumed (with efficiency increasing across immaturity), where foods are located in the home range, and the calorie costs of travelling to food sources, etc.

Given these challenges, we designed our model to focus on the key question at hand: what can immatures learn – both with and without different forms of social learning - irrespective of whether resultant repertoire size influenced survival? Restricting our model in this way allowed us to answer our target question (how does social learning influence broad-scale diet repertoire development), without making assumptions surrounding how diet size influences survivability that could not be supported by existing data. This is an additional reason why the results of our model are likely highly conservative, as it remains to be seen whether immatures could even cultivate diets which permit survival without social learning. We strongly recommend that future research addresses this interesting additional question where and when the additional required data permits.

There is no transfer of 'know-how' between food items

It is possible that wild orangutans can transfer knowledge about how to process a familiar food item onto an unknown, yet similar food (henceforth referred to as '*know-how transfer*'). However, how often know-how transfer occurs during orangutan diet learning has not been explicitly investigated. When encountering unfamiliar food items, we did not permit simulated immatures to perform know-how transfers between similarly processed foods because:

[1] understanding if and when immatures transfer know-how from a familiar food to an unfamiliar food is not discernible from our available data from the wild.

[2] very simple foods (complexity level 0) required only 1 exploration. These simple foods had the greatest likelihood of having 'know-how' transferred between food items (e.g. eating leaves from different trees is often mechanically identical). Yet, immatures would still likely require at least one exploration for individuals to learn 'what' to eat. Know-how transfers are therefore unlikely to be able to further reduce the number of explorations needed to add these foods to the diet repertoire.

[3] increasingly complex foods (such those in as complexity levels 3-5) involve progressively more processing steps. With each increasing number of steps, it becomes less likely that another food requires an identical processing behavior. Thus, when encountering unfamiliar foods which are increasingly challenging to eat, it becomes progressively less likely that orangutans would know of other foods with highly-similar processing behaviors. Indeed, even for adult male orangutans migrating into new territories, males peer more frequently at unfamiliar foods that are more difficult to process, suggesting that 'know-how' for processing these foods cannot be easily predicted from adult males' existing knowledge[14].

[4] Most importantly, if processes of know-how transfer between food items occurs in the wild, we likely already account for this implicitly when estimating the number of times wild immatures must explore food items before learning their required processing behaviors. When estimating this minimum number of explorations required to learn how to eat foods of each complexity category, we estimated the earliest age immatures were observed consuming foods, the likely number of encounters with food items that occurred by this age, and subsequently, the likely number of times immatures explored this food item prior to learning (averaging up to the 5 most common foods of each complexity category; see Methods). If transfer of 'know-how' occurred between familiar and unfamiliar food items in the wild, thus meaning foods were added to immatures' diet repertoires at earlier ages, we would have implicitly captured this process when estimating the first age at which particular food items were first observed being consumed. This would consequently lead to lower estimates for the number of times immatures encountered each food prior to learning how to consume them, and in extension, this would reduce our estimate for the number of times a food item was explored by an immature before it was added to their diet repertoire. Therefore, if know-how transfers occur in the wild, we likely capture this implicitly within our estimates.

Simulated immatures have perfect memory

Simulated immatures did not 'forget' any previous explorations with food items. Accurately assessing how frequently wild immatures forget information acquired from previous explorations, while potentially important[15], is extremely challenging, and unlikely to be possible from our dataset. However, much like our discussion of know-how transfers, the possibility that immatures forget knowledge from previous explorations may be implicitly accounted for when we estimated the number of explorations immatures require to learn how to process different foods. If wild immatures forget information learnt from previous explorations, they may have to explore more frequently prior to successfully learning. This would likely be

implicitly included in our estimate of the number times immatures explored food items prior to successfully learning how to consume different foods. However, accurately validating whether or not this is the case is challenging.

We believe that this is a reasonable simplification of our model. Additionally, the possibility of forgetting information acquired through explorations may further intensify the need for more frequent exploring during immaturity, and in extension, the need for social learning as a mechanism to upregulate and guide exploration behaviors. As such, the perfect memory of simulated immatures may present another reason why our estimation of the relative importance of different social learning mechanisms are conservative. Further research will be required to fully understand the relationships between social learning, information acquisition, and memory.

Our ABM is configured using data from a single orangutan population.

Our model was calibrated on data from a single orangutan population: Suaq. Adult orangutans from other populations exhibit lower rates of sociality than at Suaq[13], so their immatures may be under greater pressure to acquire adult-like diet repertoires by independence. Diet size also varies somewhat across populations (but habitually reaches hundreds of different known food items[16]), and diets can vary in their composition including the frequency of foods with complex processing behaviors[13]. Further studies could use comparative data across sites (and species) to characterize how these factors modulate the adaptive benefits of different forms of social learning for diet development, using similar data-driven simulation approaches.

Section 2: Statistical analyses

The formula for each statistical model referred to in our manuscript, and a brief summary of its output, can be found in Tables S2-S5. Our ABM was programmed in Python[17] (v. 3.8.11), and random numbers were generated in our ABM using the NumPy package (v. 1.22.4)[18].

2.1 Estimating parameters using data from wild orangutans at Suaq

All analyses of data from wild individuals were conducted in R (v 4.5.1 Great Square Root[19]). Poisson and binomial GLMMs were constructed using the lme4 package's glmer() function[20] (v. 1.1.37). Quasibinomial GLMMs were fit by Penalized Quasi-Likelihood using the MASS package[21](v. 7.3.65). Confidence intervals around model estimates were calculated using the parameters package (v. 0.26.0).

Feeding patches visited per day (Poisson GLMM).

A Poisson GLMM was used to model the number of feeding patches visited by mothers per day, as feeding patches were measured using discrete count data. This model was used to estimate the mean number of feeding patches visited across all follows, whilst also accounting for repeated measures from the same individual across different follows. Therefore, we did not fit a slope to our model; rather, the model was fit to an intercept only. Given that we had no dependent variable (only an intercept), testing of model dispersion was not required. Focal ID was included as a random factor to account for data sampled from the same individual over multiple follows. We restricted our analysis to follows which were at least 10 hours long, as

these follows likely covered all feeding patches visited by mothers across a day ($N_{Mothers} = 13$; $N_{Follows} = 252$; mean follow duration = 11 hours 32 minutes; SD = 41 minutes).

The probability of being in association, close/distant association, or peering across development, and the effect of peering on exploration probability across development (Binomial and Quasibinomial GLMMs)

Much of the data we modelled to calibrate our ABM was in the form of Boolean Y/N variables, and due to this fact, we modelled this data using binomial and quasibinomial GLMMs. This included all data that described the probability of being in association; the probability of being in either close or distant association; the probability of peering; and several models that evaluate the effect of peering on the probability of exploration.

Before evaluating how peering influenced exploration rates over development, we first evaluated whether peering led to higher baseline levels of exploration across all ages. When evaluating whether peering was related to the probability of exploration – regardless of an individual's age – we fit two models: one which estimated the probability that immatures explored a food item in the hour *after* peering at any foods of the same species (in instances where immatures had not already explored with the food item prior to peering), and one which estimated the probability immatures explored a food item in the hour *before* peering at any foods of the same species. This allowed us to make a direct comparison of the effect of peering on exploration rates in similar contexts (i.e. when food items that could be the target of peering and exploration were available). For both instances, if at least one exploration behavior was performed within the relevant time interval, we marked exploration as a 'success', otherwise, the performance of an exploration behavior was marked as a 'failure'. To model the probability of peering on exploration in the hour before (model 1) and the hour after (model 2), we used binomial GLMMs that fit a random intercept for the proportion of exploration behaviors performed in either context. The ID of the focal individual was included in our model as a random intercept to account for multiple samples taken from the same individuals over time. Similarly to our model of the number of feeding patches mothers encounter per day, we did not run dispersion testing on these binomial models as they fit intercepts only.

For datasets describing the rate at which immatures were in different social states during development (association, close association, and peering) and the dataset describing the effects of peering on exploration across development, we initially fit binomial models using immature age as an independent variable, and the probability of a given outcome (i.e. a given social state, or exploration) as the dependent variable. Across all of these models, Focal ID was included as a random intercept to account for sampling the same individuals across multiple follows (scans from the same follow were automatically controlled for by the model). As both successes and failures were fed to models using Boolean data, the proportion of successes and failures was automatically scaled and weighted to the length of each follow.

As binomial models that are fit to data from sampled successes and failures tend to be overdispersed, we tested for overdispersion of our binomial models using both visual inspection of the distribution of model residuals, and the testDispersion() function of the DHARMa package[22] (v. 0.4.7). We used this joint approach as the null hypothesis for dispersion tests is that there is no overdispersion, and they are therefore primed to only identify overdispersion in extreme instances. Consequently, when used in isolation, dispersion tests can miss opportunities to improve model fits where more moderate overdispersion is present.

The model describing the effect of peering on exploration over development exhibited acceptable dispersion levels (dispersion = 1.02; $p = 0.8$). However, the binomial GLMM for the association distance over development was significantly overdispersed (dispersion = 1.2; $p <$

0.001). Moreover, visual inspection of residuals for the binomial models describing the rate of association, and the probability of peering, suggested that model fit may be improved using alternative data distributions, despite not meeting the criteria of significance, $p < 0.05$ (rate of association: dispersion = 1.1, p = 0.66; peering probability: dispersion = 1.4, p = 0.38). Therefore, for both of these models, we deemed it likely that alternative distributions, such as quasibinomial model, would improve model fit, despite divergence of the data from model predictions being reasonably smaller. We therefore refit all three of these models as quasibinomial GLMMs, with identical fixed and random effects.

The probability of exploration across development (Binomial GAMM)

To analyze age-dependent exploration rates when immatures were in close and distant association, generalized additive mixed models (GAMMs) were used to estimate flexible non-linear effects during development, and were fit using the mgcv package (v.1.9.3)[23]. Exploration data in each association condition were recorded using Boolean successes and failures, and were thusly modelled using a binomial distribution. Similarly to our other models, Focal ID was included as a random intercept to avoid pseudoreplication. We modelled exploration rates over development in each association proximity separately using this model, by including a factor of two levels: **close** and **distant** association**.** As data describing exploration rates in each association proximity (close/distant) can stem from the same follow (and therefore are not always independently sampled), we included the follow number as a random factor in our model. Similarly to our other binomial and quasibinomial models, the total number of successes and failures (i.e. the total number of scans in each association proximity condition for each follow) was automatically accounted for when estimating probabilities of exploration.

Our model reached full convergence, and explained 91% of deviance, with an adjusted R^2 = 0.781. These values confirm that our model established a good fit to our data. We confirmed that there were a suitable number of basis functions to explain the relationship between age and exploration rate (in both association proximity settings) using the *gam.check()* function.

2.2 ABM Output

Analyzing model outputs using Poisson GLMs

When modelling repertoire size across experimental treatments, we used a Poisson GLM with simulation type as a categorical variable, and the number of food items in the simulated immature's repertoire as a count variable. This protocol was applied identically for repertoire sizes at the onset of independence, and at the end of immaturity. We checked for overdispersion in these Poisson models using the *check_overdispersion()* function of the Performance package[24] (v.0.14.0), for which we found no evidence (onset of independence: dispersion ratio = 0.140, Pearson's χ^2 = 104.6, p ≈ 1; end of immaturity: dispersion ratio = 0.138, Pearson's χ^2 = 103.1, p ≈ 1).

A note on ABM validation

We validated our ABM via 'output validation'. As mentioned in the main manuscript, we observed whether the treatment '*Exposure, Enhancement & Peering*' produced a trajectory for long-term diet learning which matches what is observed in wild individuals. We expected this treatment to best match diet development in wild individuals, given that previous studies have identified short-term effects of peering and enhancement on food exploration in wild

orangutans (though, these effects have never been evaluated in the long-term; see Introduction).

Given that the outcomes of the *Exposure, Enhancement & Peering* treatment were similar to those observed for wild individuals, this provided us with a key form of output validation for our model. We could then remove forms of social learning to quantify the extent to which they affect long-term diet learning across development.

If we had not reached output validation, our model could still offer key insights into the effects of social learning on diet development. For example, if the *Exposure, Enhancement & Peering* treatment substantially deviated from the outcomes observed in wild individuals (either in the age at which adult-like diets emerged, or the diet size at the onset of independence), we could have rescaled our model accordingly. During rescaling, the ABM output for the *Exposure, Enhancement & Peering* could be taken as a 'full repertoire including social learning', and the outputs of treatments when social learning was removed could have been compared to this baseline (e.g. as a % reduction in repertoire size at the onset of independence).

Thus, output validation is not necessary for an ABM to be useful in the contexts of our study. However, our output validation offered two key advantages: [1] output validation provides further confidence that we captured the majority of key processes that influence diet learning in real life, and [2] we could estimate the long-term effects of social learning directly on time-scales (and scales of diet size) that are relevant to wild orangutans.

Section 3: Supplementary Tables

Table S1. ABM coefficients for the probability of simulated immatures entering each social state when in a given feeding patch, and their subsequent probability of exploring, at different ages. All values are reported up to 3.s.f. Social states are nested, and therefore the probability of a simulated immature being in a particular social state is contingent upon their higher-level states, i.e. to be in *close association*, a simulated immature must be *in association*. To peer, a simulated immature must be in *close association*.

Simulated Age (Years)	Social State Probability			Exploration Probability		
	In Association	Close Association	Peering	Distant Association (Including alone)	Close Association	Following Peering
0	0.998	0.975	0.0303	0.00448	0.092	0.659
1	0.997	0.966	0.0270	0.0561	0.103	0.686
2	0.995	0.953	0.0240	0.0690	0.118	0.711
3	0.993	0.935	0.0214	0.0163	0.0601	0.736
4	0.990	0.911	0.0191	0.0142	0.0606	0.759
5	0.985	0.879	0.0170	0.0152	0.0451	0.780
6	0.978	0.838	0.0151	0.00584	0.0242	0.801
7	0.967	0.787	0.0134	0.00303	0.00898	0.819
8	0.952	0.724	0.0119	0.00160	0.00129	0.837
9	0.930	0.652	0.0106	0.00189	0.000342	0.853
10	0.899	0.571	0.00945	0.00156	0.00106	0.867
11	0.857	0.487	0.00840	0.00125	0.00213	0.881
12	0.801	0.403	0.00747	0.000652	0.00288	0.893
13	0.730	0.325	0.00664	0.000174	0.00263	0.904
14	0.646	0.255	0.00590	0.0000208	0.000777	0.914

Table S2. Summaries of models characterizing the foraging behaviors of adult orangutans at Suaq, including mothers. All values provided to 3.s.f., other than sample sizes and df which are presented as exact values.

Foraging behaviors of mothers and other adults

Feeding patches visited by mothers each day (intercept-only Poisson GLMM)

glmer(Patches Visited ~ 1 + (1|FocalID), family = Poisson())

* Data was filtered to only include follows which were at least 10 hours long, thus likely covering all of wild mothers' daily feeding stops.

$N_{Follows}$= 252; N_{Focals} = 13; df.resid = 250;

Fixed Effects	Intercept = 3.29 $CI_{95\%}$ [3.14,3.43]	SE = 0.0733	Z = 44.8	p < 0.001	
Random Effects (FocalID)	Var = 0.0567	SD = 0.238			

Adult repertoire size (Non-linear mixed-effect model, fit to the Michealis-Menten Equation).

- This model estimates a plateau point (Vmax) for the total number of food items adults are observed eating over cumulative sampling effort. K is the midpoint of the accumulation curve.
- To aid model fitting, we allowed K to vary between individuals, but assume that all adults' repertoire sizes will be similar. We then account for the fact that adults' diet breadths may vary somewhat, by comparing simulated diet-repertoire development to a slightly smaller threshold (90%).

```
nlme(cumulativeFoodItems~ (Vmax)*sampledScans/(K+sampledScans),
        start = c(Vmax = 250, K = 20000),
        fixed =  Vmax + K ~ 1,
        random = K  ~ 1,
        control = (msMaxIter = 20000000))
```

$N_{ScansObserved}$ = 402,082; N_{Focals} = 95

Fixed Effects	Vmax = 248	SE = 0.08	DF = 401,986	t = 2930	p < 0.001
	K = 13100	SE = 707	DF = 401,986	t = 18.5	p < 0.001
Random Effects (Focal ID)	SD: K = 6730	SD: Residual = 6.28			

Table S3. Summaries of models used to estimate the probability of immatures being in different social states across development. All values provided to 3.s.f., other than sample sizes which are presented as exact values.

**Probability of being in each social state across development**					
**Association rate across development (Quasibinomial GLMM)**<br>glmmPQL(cbind(scans_associated, scans_alone) ~ Age, random=~1|FocalID, family= "quasibinomial")<br><br>$N_{ScansObserved}$ = 99,264; $N_{Follows}$ = 362; N_{Focals} = 30.					
Fixed Effects	Intercept = 6.15	SE = 0.718	DF = 331	t = 8.57	p < 0.001
	Age = -0.397;<br>$CI_{95\%}$ [-0.51,-0.28]	SE = 0.0594	DF = 331	t = -6.68	p < 0.001
Random Effects (FocalID)	SD: Intercept = 1.88	SD: Residual = 7.39			
**Close association rate across development (Quasibinomial GLMM)**<br>glmmPQL(cbind(scans_closeAssociation, scans_distantAssociation) ~ Age, random=~1|FocalID, family= "quasibinomial")<br><br>$N_{ScansObserved}$ = 78,478, $N_{Follows}$ = 323; N_{Focals} = 28					
Fixed Effects	Intercept = 3.68	SE = 0.259	DF = 294	t = 14.2	p < 0.001
	Age = -0.339<br>$CI_{95\%}$ [-0.4,-0.28]	SE = 0.0302	DF = 294	t = -11.2	p < 0.001
Random Effects (FocalID)	SD: Intercept = 0.484	SD: Residual = 6.60			
**Peering rate across development (Quasibinomial GLMM)**<br>glmmPQL(cbind(scans_Peering, scans_notPeering) ~ Age, random=~1|FocalID, family= "quasibinomial")<br><br>$N_{ScansObserved}$ = 66,501; $N_{Follows}$ = 311; N_{Focals} = 28					
Fixed Effects	Intercept = -3.47	SE = 0.200	DF = 282	t = -17.4	p < 0.001
	Age = -0.119<br>$CI_{95\%}$ [-0.17,-0.07]	SE = 0.0263	DF = 282	t = -4.52	p < 0.001
Random Effects (FocalID)	SD: Intercept = 0.634	SD: Residual = 2.08			

Table S4. Summaries of models used to characterize the relationships between immatures' ages, social states, and their resultant effects on the probability of exploration. All values provided to 3.s.f., other than sample sizes which are presented as exact values.

Effects of social states on exploration

Effect of association proximity on exploration probability across development (Binomial GAMM)

```
gam(cbind(scans_Exploring, scans_notExploring) ~ s(age, k = 10, m = 2) +
          s(Age, closeAssociation, bs = 'fs') +
          s(Age, focalID, bs = 're') +
          s(Age, followNr, bs = 're'),
          family = binomial('logit'),
          data = expsum,
          method = 'REML')
```

$N_{ScansObserved}$ = 81,153; across two groups (close/distant association 10 m). N_{Focals} = 30; $N_{Follows}$ = 364

Smooth terms	s(Age)	edf = 3.02	Ref.df = 3.16	X^2 = 18.7	p = 0.00138
	s(Age, closeAssociation)	edf = 9.54	Ref.df = 18	X^2 = 1770	p < 0.001
	s(Age, focalID)	edf = 16.8	Ref.df = 27	X^2 = 9286	p = 0.00428
	s(Age, followNr)	edf = 202	Ref.df = 340	X^2 = 6580	p < 0.001

Exploration probability in the hour before peering (intercept-only Binomial GLMM)

```
glmer(Explore_BeforePeering ~ 1 + (1|FocalID), family = binomial())
```

$N_{Observations}$ = 367; N_{Focals} = 14

Fixed Effects	Intercept = -0.685	SE = 0.184	Z = -3.72	p < 0.001	
Random Effects (FocalID)	Var = 0.115	SD = 0.339			

Exploration probability in the hour following peering (intercept-only Binomial GLMM)

```
glmer(Explore_AfterPeering ~ 1 + (1|FocalID), family = binomial())
```

*Data excludes any observations where immatures explored prior to peering.

$N_{Observations}$ = 238; N_{Focals} = 13

Fixed Effects	Intercept = 0.742	SE = 0.175	Z = 4.24	p < 0.001	
Random Effects (FocalID)	Var = 0.0421	SD = 0.205			

Effect of peering on exploration probability across development (Binomial GLMM)

```
glmer(Explore_AfterPeering ~ Age + (1|FocalID), family = binomial(link = 'logit'))
```

$N_{Observations}$ = 367; N_{Focals} = 14

Fixed Effects	Intercept = 0.658	SE = 0.198	Z = 3.33	p < 0.001	
	Age = 0.122<br>$CI_{95\%}$ = [0.03,0.22]	SE = 0.0475	Z = 2.57	p = 0.0102	
Random Effects (FocalID)	Var = 0.0385	SD = 0.196			

Table S5. Summaries of models used to characterize the outputs of the ABM. The first two models characterize simulated immatures' diet-repertoire breadths at key milestones across the three experimental treatments. The final model compares the ages at which adult-like diet repertoires emerged in simulated immatures, compared with the age at which wild immatures became independent from their mothers. All values provided to 3.s.f., other than sample sizes which are presented as exact values.

**Analyzing ABM Outputs**				
**Diet-repertoire size at the maximum age of independence, 9 years (Poisson GLM)**<br>glm(Repertoire_Size9Years ~ Simulation, data, family = Poisson)<br><br>$N_{observations}$ = 750, across 3 treatments (250 each).				
Fixed Effects	Intercept = 5.41<br>$CI_{95\%}$ [5.4,5.42]	SE = 0.00423	Z = 1280	$p < 0.001$
	Exposure & Social Enhancement = -0.0504<br>$CI_{95\%}$ [-0.06,-0.04]	SE = 0.00606	Z = -8.32	$p < 0.001$
	Exposure Only = -0.388<br>$CI_{95\%}$ [-0.40,-0.37]	SE = 0.00665	Z = -58.3	$p < 0.001$
**Diet-repertoire size at the end of immaturity, 15 years (Poisson GLM)**<br>glm(Repertoire_Size15Years ~ Simulation, family = Poisson)<br><br>$N_{observations}$ = 750, across 3 treatments (250 each).				
Fixed Effects	Intercept = 5.42	SE = 0.00421	Z = 1290	$p < 0.001$
	Exposure & Social Enhancement = -0.0559<br>$CI_{95\%}$ [-0.07,-0.04]	SE = 0.00604	Z = -9.25	$p < 0.001$
	Exposure Only = -0.385<br>$CI_{95\%}$ [-0.40,-0.37]	SE = 0.00662	Z = -58.2	$p < 0.001$
**Comparing the age that adult-like diet repertoires emerged in simulated immatures, compared to the age of independence of wild immatures (LM)**<br>lm(Ages ~ SimulationOrWild)<br>*Data confined to the *Exposure, Social Enhancement and Peering* Treatment<br><br>N_{Wild} = 8, $N_{Simulated}$ = 196				
Intercept = 7.55	SE = 0.148	t(202) = 50.9	$p < 0.001$	
Wild = 0.634<br>$CI_{95\%}$ [-0.84,2.11]	SE = 0.749	t(202) = 0.847	t = 0.398	

Section 4: Supplementary references:

1. Bryson, J. J., Ando, Y. & Lehmann, H. Agent-based modelling as scientific method: a case study analysing primate social behaviour. *Phil. Trans. R. Soc. B* **362**, 1685–1699 (2007).
2. Smaldino, P. Models Are Stupid, and We Need More of Them. in *Computational Social Psychology* (eds. Vallacher, R. R., Read, S. J. & Nowak, A.) 313–331 (Routledge, New York, 2017).
3. Marzec, A. Innovations and social barriers in orangutan culture. (University of Zurich, Zurich, Switzerland, 2020).
4. Russon, A. E. Return of the Native: Cognition and Site-Specific Expertise in Orangutan Rehabilitation. *Int. J. Primatol.* **23**, 461–478 (2002).
5. Beck, B. *et al.* Losses and reproduction in reintroduced golden lion tamarins *Leontopithecus rosalia*. *Dodo* **27**, 50–61 (1991).
6. Ehmann, B. *et al.* Immature wild orangutans acquire relevant ecological knowledge through sex-specific attentional biases during social learning. *PLoS Biol.* **19**, e3001173 (2021).
7. Schuppli, C., Van Cauwenberghe, A., Mirta Setia, T. & Haun, D. The ontogeny of exploratory object manipulation behaviour in wild orangutans. *Evolut. Hum. Sci.* 1–32 (2021) doi:10.1017/ehs.2021.34.
8. Schuppli, C. *et al.* Observational social learning and socially induced practice of routine skills in immature wild orang-utans. *Anim. Behav.* **119**, 87–98 (2016).
9. Laumer, I. *et al.* Wild and zoo-housed orangutans differ in how they explore the world: comparing object manipulations in Sumatran orangutans. *Sci. Rep.* **15**, 14853 (2025).
10. Kunz, J. A. *et al.* Play Behavior Varies with Age, Sex, and Socioecological Context in Wild, Immature Orangutans (Pongo spp.). *Int. J. Primatol.* **45**, 739–773 (2024).
11. Kukofka, P. *et al.* The development of social attention in orangutans: Comparing peering behavior in wild and zoo-housed individuals. *iScience* **28**, 111542 (2025).
12. Barrett, B. J., McElreath, R. L. & Perry, S. E. Pay-off-biased social learning underlies the diffusion of novel extractive foraging traditions in a wild primate. *Proc. R. Soc. B* 1–10 (2017).
13. Schuppli, C. *et al.* The effects of sociability on exploratory tendency and innovation repertoires in wild Sumatran and Bornean orangutans. *Sci. Rep.* **7**, 15464 (2017).
14. Mörchen, J. *et al.* Migrant orangutan males use social learning to adapt to new habitat after dispersal. *Front. Ecol. Evol.* **11**, 1158887 (2023).
15. Ammar, M., Fogarty, L. & Kandler, A. Social learning and memory. *Proc. Natl. Acad. Sci. U.S.A.* **120**, e2310033120 (2023).
16. Russon, A. E. *et al.* Geographic variation in orangutan diets. in *Orangutans: Geographic Variation in Behavioral Ecology and Conservation* (eds. Wich, S., Suci Utami Atmoko, S., Mitra Setia, T. & Van Schaik, C. P.) 135–156 (Oxford University Press, 2009).
17. Van Rossum, G. & Drake, F. L. Python 3 Reference Manual. CreateSpace (2009).
18. Harris, C. R. *et al.* Array programming with NumPy. *Nature* **585**, 357–362 (2020).
19. R Core Team. R: A language and environment for statistical computing. R Foundation for Statistical Computing (2022).
20. Bates, D., Mächler, M., Bolker, B. & Walker, S. Fitting Linear Mixed-Effects Models Using **lme4**. *J. Stat. Soft.* **67**, (2015).
21. Venables, W. & Ripley, B. *Modern Applied Statistics with S*. (Springer New York, 2002).
22. Hartig, F. DHARMa: Residual Diagnostics for Hierarchical (Multi-Level / Mixed) Regression Models. (2022).
23. Wood, S. N. Fast Stable Restricted Maximum Likelihood and Marginal Likelihood Estimation of Semiparametric Generalized Linear Models. *Journal of the Royal Statistical Society Series B: Statistical Methodology* **73**, 3–36 (2011).

24. Lüdecke, D., Ben-Shachar, M. S., Patil, I., Waggoner, P. & Makowski, D. performance: An R Package for Assessment, Comparison and Testing of Statistical Models. *J. Open Source Softw.* **6**, 3139 (2021).
