# Supplementary material for: Culture is critical in driving orangutan diet development past individual potentials
Source: Nat Hum Behav. 2025 Nov 24;10(2):243–54. doi: 10.1038/s41562-025-02350-y (PMC12932104; doi:10.1038/s41562-025-02350-y)
Supplement: Supplementary file 2 — Reporting Summary [file 41562_2025_2350_MOESM2_ESM.pdf]

nature portfolio

Corresponding author(s): Elliot Howard-Spink, Caroline Schuppli

Last updated by author(s): 29.09.2025

Reporting Summary

Nature Portfolio wishes to improve the reproducibility of the work that we publish. This form provides structure for consistency and transparency in reporting. For further information on Nature Portfolio policies, see our Editorial Policies and the Editorial Policy Checklist.

Statistics

For all statistical analyses, confirm that the following items are present in the figure legend, table legend, main text, or Methods section.

n/a	Confirmed	
☐	☒	The exact sample size (n) for each experimental group/condition, given as a discrete number and unit of measurement
☐	☒	A statement on whether measurements were taken from distinct samples or whether the same sample was measured repeatedly
☐	☒	The statistical test(s) used AND whether they are one- or two-sided<br>*Only common tests should be described solely by name; describe more complex techniques in the Methods section.*
☐	☒	A description of all covariates tested
☐	☒	A description of any assumptions or corrections, such as tests of normality and adjustment for multiple comparisons
☐	☒	A full description of the statistical parameters including central tendency (e.g. means) or other basic estimates (e.g. regression coefficient) AND variation (e.g. standard deviation) or associated estimates of uncertainty (e.g. confidence intervals)
☐	☒	For null hypothesis testing, the test statistic (e.g. F, t, r) with confidence intervals, effect sizes, degrees of freedom and P value noted<br>*Give P values as exact values whenever suitable.*
☒	☐	For Bayesian analysis, information on the choice of priors and Markov chain Monte Carlo settings
☐	☒	For hierarchical and complex designs, identification of the appropriate level for tests and full reporting of outcomes
☐	☒	Estimates of effect sizes (e.g. Cohen's d, Pearson's r), indicating how they were calculated

Our web collection on statistics for biologists contains articles on many of the points above.

Software and code

Policy information about availability of computer code

Data collection	No custom code was used for data collection.
Data analysis	All data analysis was conducted in R (v 4.5.1 Great Square Root). We used GAMM and GLMM models to analyze data collected from wild individuals. Poisson and binomial GLMMs were constructed using the lme4 package's glmer() function (v. 1.1.37). Quasibinomial GLMMs were fit by Penalized Quasi-Likelihood using the MASS package (v. 7.3.65). GAMMs were fit using the mgcv package (v.1.9.3). Confidence intervals around model estimates were calculated using the parameters package (v. 0.26.0) For binomial models, dispersion tests were run using the testDispersion() function of the DAHRMa package (v 0.4.7). Dispersion of Poisson models were checked using the check_overdispersion() function of the Performance package (v. 0.14.0). A detailed account of the design of all models is provided within the Supplementary Information. Our ABM was programmed using Python (v. 3.8.11), and random numbers were generated using NumPy (v. 1.22.4). All code used for data analysis and to run the ABM can be retrieved using the link under 'Data and Code Availability' within the main manuscript. Versions for all packages and software are included in the supplementary materials.

For manuscripts utilizing custom algorithms or software that are central to the research but not yet described in published literature, software must be made available to editors and reviewers. We strongly encourage code deposition in a community repository (e.g. GitHub). See the Nature Portfolio guidelines for submitting code & software for further information.

Data

Policy information about availability of data

All manuscripts must include a data availability statement. This statement should provide the following information, where applicable:

- Accession codes, unique identifiers, or web links for publicly available datasets
- A description of any restrictions on data availability
- For clinical datasets or third party data, please ensure that the statement adheres to our policy

All data supporting our results can be found in the following repository: https://data.mendeley.com/datasets/7kvr22vk5f/3

Research involving human participants, their data, or biological material

Policy information about studies with human participants or human data. See also policy information about sex, gender (identity/presentation), and sexual orientation and race, ethnicity and racism.

Reporting on sex and gender	N/A
Reporting on race, ethnicity, or other socially relevant groupings	N/A
Population characteristics	N/A
Recruitment	N/A
Ethics oversight	N/A

Note that full information on the approval of the study protocol must also be provided in the manuscript.

Field-specific reporting

Please select the one below that is the best fit for your research. If you are not sure, read the appropriate sections before making your selection.

☐ Life sciences ☐ Behavioural & social sciences ☒ Ecological, evolutionary & environmental sciences

For a reference copy of the document with all sections, see nature.com/documents/nr-reporting-summary-flat.pdf

Ecological, evolutionary & environmental sciences study design

All studies must disclose on these points even when the disclosure is negative.

Study description	We leveraged long term data collected at the Suaq Balimbing research area, Sumatra Indonesia. Data was collected on the behaviors of wild Sumatran orangutans (Pongo abelii). All individuals included in this study are well known, and form part of long-term data collection at Suaq.
Research sample	Our dataset on wild orangutans included data collected from 2676 focal follows on 132 individuals, totaling 22,547 hours of observation time. Each analysis within our manuscript used a different subset of this data based on the data's relevance. For example, when estimating adult diet repertoire size we only used data from individuals who had reached sexual maturity (Adults = 95; Follows = 1620; Scans = 402,082). We provide a full list of sample sizes for each analysis (including at different levels of the data where relevant, such as the number of individuals from whom we collected data for a given analysis, and the total number of behavioral scans sampled across these individuals).
Sampling strategy	Within the forest, focal orangutans were followed opportunistically upon encounter. Once encountered, orangutans continued to be followed throughout the day until they made night nests. Orangutans were followed for a maximum of 10 consecutive days, after which another individual was saught out for sampling. Our data is therefore both long-term and cross-sectional, as not all individuals can be followed on each day of data collection. We sampled and analyzed all data collected between 2007-2019.
Data collection	Data was recorded on paper or digital tablets. Data was recorded on focal orangutan behaviors at 2-minute intervals by a trained team of researchers and field assistants via instantaneous sampling. Instantaneous sampling recorded all behaviors performed by orangutans, as well as any objects or food items associated with these behaviors. These behaviors included all instances of exploration (including solo object play, and failed feeding attempts) as well as feeding and peering behaviors. Additional data was collected on peering behaviors in wild orangutans on an all-occurence basis by one observer (C.S.) who specifically collected data on how peering behaviors related to exploration of target food items in the hour before and after peering. In both sampling strategies, the target of orangutans' observations during peering (including the individual being peered at, the target individual's behavior, and any objects they are manipulating) were recorded recorded. A link to the full protocol for data collection at Suaq is provided as part of our online methods.

Timing and spatial scale	We sampled and analyzed all data collected between 2007-2019. All data was recorded at Suaq Balimbing Monitoring Station in South Aceh, Sumatra, Indonesia, which covers 550ha, at around 5m above sea level.
Data exclusions	We only included data from observers who passed minimum thresholds of inter-observer reliability. For each analysis, we only used data which was relevant to the question at hand (see example above on adult diet-repertoire size). Any data which was filtered for each analysis is clearly described within the methods and results of our manuscript, including sample sizes after filtering.
Reproducibility	N/A, data were collected through day-long observations of wild animals.
Randomization	N/A, data were collected through day-long observations of wild animals, without interacting with the animals, or assigning them to any experimental 'treatments'.
Blinding	N/A, the animals were individually known to the observers, and this was a fundamental aspect of long-term data collection at the individual level at Suaq.

Did the study involve field work? ☒ Yes ☐ No

Field work, collection and transport

Field conditions	Data was collected at the Suaq Balimbing Monitoring Station in South Aceh, Sumatra, Indonesia. This research area covers a deep peat swamp forest. There is standing water covering the area year round, which raises during wetter seasons (two distinct periods per year). At the centre of the research area, water can reach chest height. Temperatures range from 19-38 degrees celsius each day.
Location	Data was collected at the Suaq Balimbing Monitoring Station in South Aceh, Sumatra, Indonesia (3° 02.873′ N, 97° 25.013′E). This research area covers 550ha and is approximately 5m above sea level.
Access & import/export	This field site can be accessed by boat. via the Lembang River. No physical samples were collected during this study.
Disturbance	No disturbance was experienced during the study period. All observers take specific care not to disturb orangutans during observation. Given that orangutans are arboreal, observers always maintain a suitable distance from observed individuals.

Reporting for specific materials, systems and methods

We require information from authors about some types of materials, experimental systems and methods used in many studies. Here, indicate whether each material, system or method listed is relevant to your study. If you are not sure if a list item applies to your research, read the appropriate section before selecting a response.

Materials & experimental systems

n/a	Involved in the study	
☒	☐	Antibodies
☒	☐	Eukaryotic cell lines
☒	☐	Palaeontology and archaeology
☐	☒	Animals and other organisms
☒	☐	Clinical data
☒	☐	Dual use research of concern
☒	☐	Plants

Methods

n/a	Involved in the study	
☒	☐	ChIP-seq
☒	☐	Flow cytometry
☒	☐	MRI-based neuroimaging

Animals and other research organisms

Policy information about studies involving animals; ARRIVE guidelines recommended for reporting animal research, and Sex and Gender in Research

Laboratory animals	No laboratory animals were used in our study.
Wild animals	We collected observational data on a population of wild Sumatran orangutans (Pongo abelii) which are habituated to human presence since the inception of research in 2007. No animals were captured or transported as part of this study. All data was collected non-invasively through observation. The age of animals observed varied from less than 1 year old to (what we estimate to be) over 60 years of age, and long term data collection includes the collection of data on both male and female animals. These animals continue to live freely in the wild.
Reporting on sex	N/A
Field-collected samples	No sampled materials were taken from this field site.

Ethics oversight	The Indonesian State Ministry for Research and Technology (RISTEKDIKTI) and the National Research and Innovation Agency (BRIN). See our Ethics Statement.

Note that full information on the approval of the study protocol must also be provided in the manuscript.

Plants

Seed stocks	No seed stocks were used in this study.
Novel plant genotypes	No novel plant genotypes were generated in this study.
Authentication	N/A
